# Mindful Learning Experience Facilitates Mastery Experience Through Heightened Flow and Self-Efficacy in Game-Based Creativity Learning

**DOI:** 10.3389/fpsyg.2019.01593

**Published:** 2019-07-17

**Authors:** Yu-chu Yeh, Szu-Yu Chen, Elisa Marie Rega, Chin-Shan Lin

**Affiliations:** ^1^College of Education, National Chengchi University, Taipei, Taiwan; ^2^Research Center for Mind, Brain and Learning, National Chengchi University, Taipei, Taiwan

**Keywords:** creativity, flow experience, mastery experience, mindful learning, self-efficacy, game-based learning

## Abstract

This study was performed within the limited framework of computer-game-based educational programs designed to enhance creativity. Furthermore, the utilization of mindful learning and moderators such as flow, mastery experience, and self-efficacy, brings this research to the forefront of modern educational practices. The present researchers developed a comprehensive game-based creativity learning program for fifth and sixth grade pupils. Further analyses presented relationship trends between mindful learning experience, flow experience, self-efficacy, and mastery experience. Eighty-three 5th and 6th grade participants undertook the six-week game-based creativity learning program. Upon completion of the experimental instruction, self-evaluation revealed that participants with higher scores on the concerned variables improved more in both creative ability and confidence than their counterparts. Additionally, path model analysis revealed that mindful learning experience was a powerful predictor of both mastery experience and flow experience; it also influenced mastery experience through flow experience and self-efficacy. The findings support the effectiveness of the game-based learning program developed in this study. Moreover, this study contributes to the theoretical construction of how game-based learning can be designed to facilitate mindful learning experience, flow experience, self-efficacy, and mastery experience during creativity. Some additional enhancement mechanisms utilized in the program were: rewards for high-quality performance, challenging tasks, a variety of design components, immediate feedback, and idea sharing. The theoretical design of this study provides support for the ongoing scientific investigation of new applications of mindful learning in educational programs concerning the learning of creativity.

## Introduction

Creativity is thought to be the development of novel ideas, or solutions to problems that benefit the larger group (Hennessey and Amabile, 2010), or the process of generating contextually or culturally original and valuable products (Yeh, 2017). Recently, there has been an influx of research identifying contextual and personal factors that foster creativity (Wang et al., 2018) as a part of the ongoing investigation into the elements of creativity. As an important catalyst for societal progress and change (Bellanca and Brandt, 2010), the current need for creativity provides evidence that creativity cultivation is a necessary component in education at all levels. Robust early education programs designed to promote creativity competence have promising rewards for future generations.

With the rise in popularity of computer or hand held device game playing, researchers are evaluating potential benefits of digital game-based learning (e.g., Hutton and Sundar, 2010; Starks, 2014; Hieftje et al., 2017; Yeh and Lin, 2018). Many studies have found that well-designed scholastic gaming environments can bolster engagement while additionally enhancing motivation and learning outcomes (e.g., Hutton and Sundar, 2010; Ahmad and Jaafar, 2012; Hung et al., 2012; Perrotta et al., 2013). Researchers have also demonstrated that computer-based games can be effective tools for motivating and engaging elementary learners for difficult subjects (Hieftje et al., 2017). Moreover, empirical evidence lends support to the concept that digital gaming increases creativity. A variety of research has shown that videogame playing positively correlates with creativity (Jackson et al., 2012), digital game-based learning systems are effective learning tools for fostering students’ creativity (Hsiao et al., 2014), and the use of problem-solving digital games can facilitate preschoolers’ creativity (Fessakis et al., 2015). Therefore, digital games can be a promising tool for improving creativity.

It has been suggested that when designing creativity training programs, a combination of cognitive, personality, motivation, and social interaction approaches should be considered (Scott et al., 2004). This study therefore developed a creativity game-based program that incorporates mechanisms to enhance mindful learning, flow experience, self-efficacy, and mastery experience in creativity. Mindful learning, which is a term developed by Langer, is an educational approach described as “the continuous creation of new categories, openness to new information, and an implicit awareness of more than one perspective” (Langer, 2016, p. 4). It is closely related to creativity because it is a divergent thinking and context-dependent approach to ideas (Nusbaum and Silvia, 2011), and some key concepts of mindfulness directly overlap with creativity (Davenport and Pagnini, 2016; Bercovitz et al., 2017). Adapting mindful practices and promoting mindful learning in game-based training may facilitate the mastery experience of creativity. In addition, mindful learning may enhance flow experience which leads to goal achievement (Kee and Wang, 2008; Reid, 2011), which may, further, carry effects on self-efficacy (Caldwell et al., 2010) and mastery experience (Bakosh et al., 2016) during game-based creativity learning.

Within the context of game-based creativity learning, this study aimed to explore how mindful learning may influence elementary school children’s mastery experience through the mediation of personal traits such as flow experience and self-efficacy. To investigate these relationships, a computer-based experimental instruction was employed and a path model was verified.

## Related Work

### Mindful Learning and Mastery Experience of Creativity in Game-Based Learning

The literature on mindfulness has focused on two principal schools of thought: one promoted by Kabat-Zinn and his associates (e.g., Kabat-Zinn, 2003), which is based on Buddhist meditation practices and often regarded as an Eastern approach to mindfulness, and the other presented by Langer and her colleagues (e.g., Langer, 1989), which is considered a Western view on mindfulness (Ivtzan and Hart, 2016). Langerian concepts serve as the backbone of the present research involving mindfulness and mindful learning. Langer and Moldoveanu (2000) defined mindfulness as the process of drawing novel distinctions, where the pertinent action is to stay in the present moment by noticing new things. This mindful behavior inspires greater sensitivity to the surrounding environment, openness to new information, concepts of new categories or perceptions, and enhanced awareness of various perspectives within problem solving (Langer and Moldoveanu, 2000; Davenport and Pagnini, 2016). Moreover, mindfulness increases flexibility pertaining to attitude, valence, perceived experience, perceived control, and self-efficacy by altering cognitive, affective, or behavioral factors (Gärtner, 2013). When encountering negative emotions, mindfulness may bolster coping abilities that help decrease negative thoughts which undermine self-efficacy (Gärtner, 2013).

More recently, Bercovitz et al. (2017) addressed that cognitive flexibility, novelty production, novelty seeking, and openness are all central components of both Langerian mindfulness and creativity. When problem solving, the mindful learner exercises divergent thinking by imagining various perspectives to find multiple solutions (Langer, 1993). These parallels between Langerian mindfulness and creativity have additionally been explored in school settings. Study findings (Davenport and Pagnini, 2016) revealed that classroom implementation of inquiry-based mindful learning strategies presented in three stages of exploration, expression and exposition, provided significant opportunities for students to exercise skills in creativity; as classrooms devoted 4 weeks to each of these three stages, teachers guided students through activities that required creativity, communication, collaboration and critical thinking. Given these empirical results, we presuppose that mindful participants may encounter mastery experience during the game-based creativity learning.

Mastery experience is the personal experience of success (Bandura, 1997). In this study, mastery experience pertains to the ability and confidence in solving problems during game-based creativity learning. Mindful learning may directly influence the process of developing mastery experience through improving the acquisition of knowledge, remaining open to feedback, and enhancing focus and awareness. These mindful learning techniques were found to be effective in improving elementary school students’ mastery experience in reading, science, and math (Anglin et al., 2008; Bakosh et al., 2016). Therefore, when mindful learning is implemented by the student, creativity may be enhanced (Davenport and Pagnini, 2016) and this improved performance is likely to contribute to the feeling of success, otherwise known as mastery experience (Bandura, 1997). The researchers of the present study drew connections between mindful learning and mastery experience to investigate possible use within digital creativity enhancement games.

### Indirect Influences of Mindful Learning on Mastery Experience

Mindful learning may contribute to the improvement of mastery experience in creativity games through flow experience and self-efficacy. Flow refers to an optimal experience in which individuals are completely absorbed or engaged in an activity (Bellanca and Brandt, 2010). There are nine elements of flow, including challenge-skill balance, action-awareness merging, clear goals, unambiguous feedback, concentration on the task at hand, sense of control, loss of self-consciousness, transformation of time, and internally-driven experience (Beard, 2015). When experiencing flow, the mind is performing at an elevated level, balancing task complexities with strategies, fully engaged, and intrinsically motivated (Csikszentmihalyi, 1990, 1993, 2014), which may result from mindful learning. It was found that when attainable goals gradually increased in difficulty, a consistent mastery curve and overall enjoyment of playing on a personal or even social level appeared (Starks, 2014). Cognitive science research also suggests that flow is achieved when technical skills are in harmony with the complexities of the task (Bellanca and Brandt, 2010) or in situations where attention is directed towards goal achievement (Reid, 2011). Accordingly, flow experience should contribute to the achievement of mastery experience.

On the other hand, self-efficacy refers to individuals’ confidence in their own abilities to execute actions with a desired outcome. People with self-efficacy act with forethought, self-reactiveness, and self-reflectiveness (Bandura, 2001); they also persevere in their goals and demonstrate resiliency to attain the desired outcome. In addition, self-efficacy provides foundations for predicting behaviors pertaining to attention, or motivational processes in learning or education (Bandura, 2012). Notably, self-efficacy can mediate the quality of products in creative endeavors pertaining to education or in the workplace (Liao et al., 2010; Wang et al., 2018). Similarly, creative self-efficacy refers to efficacy that is specific to the belief in one’s ability to produce creative outcomes (Tierney and Farmer, 2002), and it is thought to be a mechanism of creativity (Wang et al., 2018). It may also additionally reflect intrinsic motivation to exhibit creative behaviors (Gong et al., 2009).

Research has demonstrated that people who score higher on creative self-efficacy tend to be more creative (Tierney and Farmer, 2002; Gong et al., 2009; Wang et al., 2018). Creative self-efficacy has also been observed to mediate employee’s creative performance (Tierney and Farmer, 2011). This relationship between employee creative self-efficacy and creative performance has additionally been replicated in student learning. Students who scored higher on creative self-efficacy were less likely to cease efforts and disengage from a creative project or process (Liu et al., 2016). This personality characteristic that can be taught and improved upon (Tierney and Farmer, 2011) has a clear relationship to creative performance. The aforementioned research provides a basis for the current study that explores the role of creative self-efficacy in facilitating mastery experience in computer-based creativity training.

Flow and mindfulness are positively correlated (Beard, 2015; Kaufman et al., 2018) and both increase attention and focus (e.g., Bakosh et al., 2016). Athletes who scored higher in mindfulness demonstrated elevated abilities in challenge-skill balance, synthesizing action and awareness, goal setting, loss of self-consciousness, concentration, attention control, emotional control, and self-talk. These findings imply that mindfulness can amplify flow dispositions and mental skills (Kee and Wang, 2008). When utilizing mindful learning practices to bolster flow and self-efficacy, these factors are likely to improve mastery experience. Additionally, a review article on mindfulness highlighted the association between mindfulness and self-efficacy (Caldwell et al., 2010). Researchers (Greason and Cashwell, 2011) observed that mindfulness was a significant predictor of counseling self-efficacy and attention was a mediator of that relationship. In the same vein, an empirical study found that the negative influence of abusive supervision on employee self-efficacy can be buffered by employee mindfulness (Zheng and Liu, 2017). In light of the positive relationship between mindfulness, flow, self-efficacy, and learning outcomes that have been found in various domains, the current researchers presumed that there would be a positive connection between mindfulness, flow experience, self-efficacy, and mastery experience within game-based creativity learning.

### The Present Study and Hypotheses

**Figure 1:**
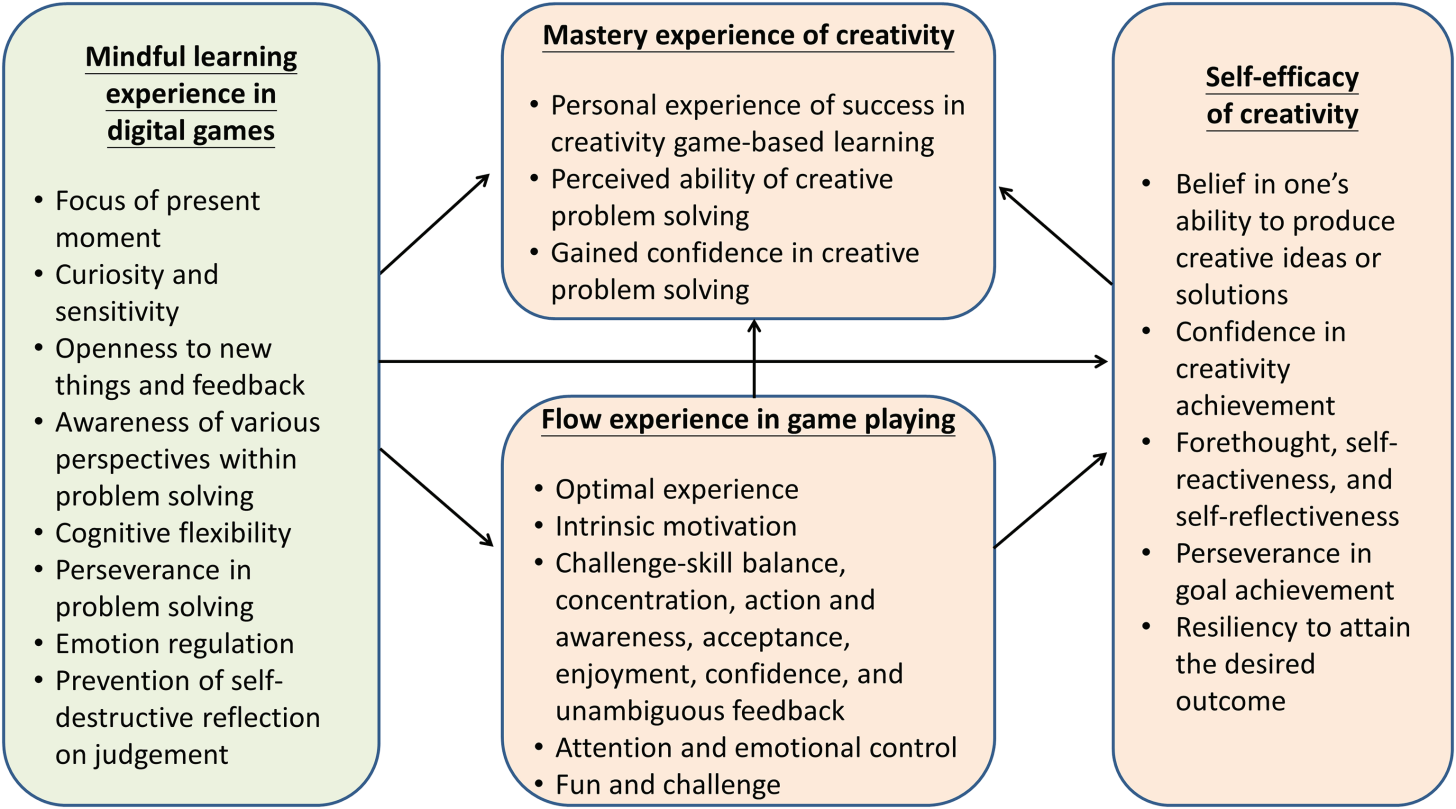
Theoretical model for proposed hypotheses.

To date, game-based learning programs that include comprehensive creativity skills and disposition training are still very limited. Based on a previously developed training program for 3rd and 4th graders (Yeh and Lin, 2018), the current program for 5th and 6th graders provides more challenging story content and tasks. This study first examined the learning effects of the developed program—Digital Game-based Learning of Creativity (DGLC-B) and, further, explored the relationship among mindful learning experience, flow experience, self-efficacy, and mastery experience. Experimental instruction was delivered and it was hypothesized that pupils would improve their creativity upon completion of the game-based creativity training; moreover, it was additionally hypothesized that mindful learning experience would directly influence mastery experience and self-efficacy, as well as indirectly influence mastery experience through flow experience and self-efficacy in the game-based creativity learning. The hypothesized theoretical model with an integrated literature review is illustrated in Figure 1.

## Materials and Methods

### Participants

The participants in this study included 39 fifth and 44 sixth graders (46 boys and 37 girls) selected through purposive sampling from four classes in an elementary school. All participants were rewarded with a Pokemon USB stick. Written informed consent was obtained from the parents of the participants in this study.

### Instruments

#### DGLC-B

**Figure 2:**
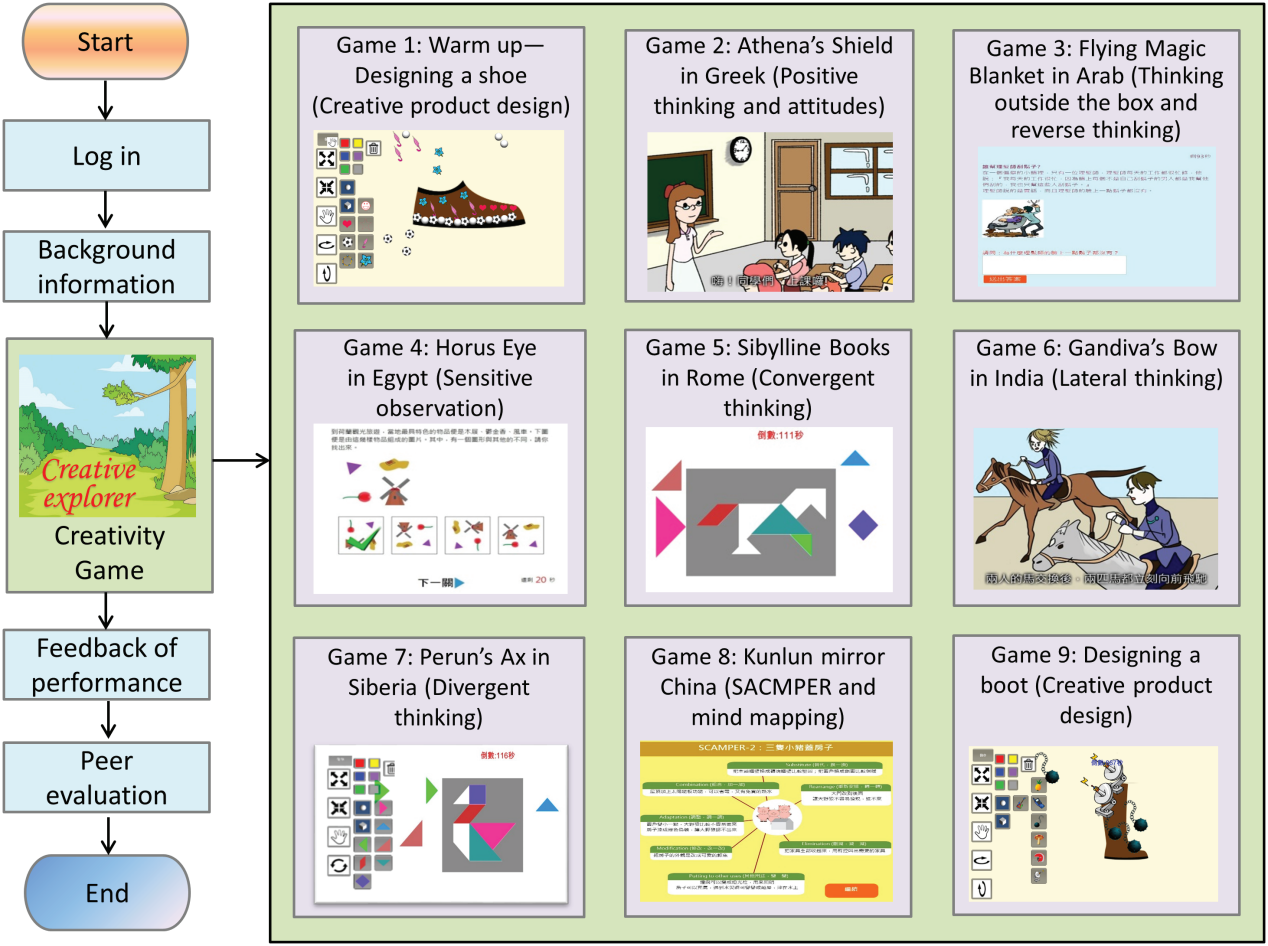
Contents and procedures of the DGLC-B.

The Digital Game-based Learning of Creativity (DGLC-B) was developed for 5th and 6th graders to investigate the participants’ learning effects and the relationship among the concerned variables. The DGLC-B, aimed to improve learners’ ability and confidence in creativity, contains a series of stories entitled “Searching for Eight Lost Treasures” based on myths from eight countries. The DGLC-B included nine games, ranging from 10 to 15 min of playing time for each game. The main contents, training focuses, and the sampled screens are illustrated in Figure 2.

The DGLC-B included the strategies for enhancing creativity dispositions of positive thinking, thinking outside the box, and reverse thinking (game 2 and game 3), as well as creativity strategies such as sensitivity in observation, convergent thinking, divergent thinking (Yeh, 2006), lateral thinking (De Bono, 1995), mind mapping (Buzan, 1996), and SCAMPER (substitution, combination, adaptation, modification, putting to other uses, elimination, and reversing) (Eberle, 1996). Finally, the DGLC-B included the activity of creative product design to allow for the implementation of creativity strategies.

#### Inventories

Four inventories were employed in this study to measure the concerned variables. They were 6-point Likert-type scales with response options ranging from “totally disagree” to “totally agree.” The Mindful Learning Experience in Digital Games (IMLE-DG) measured the participants’ experience of mindful cognition and mindful emotion during game playing. With a total of 14 items, the IMLE-DG included three factors: curiosity and open-mindedness (3 items), attention and grit (4 items), and emotion regulation (7 items). Exploratory factor analysis (EFA) revealed that the factor loadings ranged from 0.634 to 0.962, and 88.72% of the total variance was explained by the three factors. The Cronbach’s *α* coefficient was 0.974 for the IMLE-DG; the Cronbach’s *α* coefficients for the three factors were 0.947, 0.955, and 0.971 f, respectively. Confirmatory factor analysis (CFA) indicated that the IMLE-DG had good construct validity and reliability, *χ*^2^(*N* = 181, df = 56) = 119.442 (*p* < 0.001), GFI = 0.913, AGFI = 0.858, RMR = 0.073, and RMSEA = 0.079. The test items included statements such as “When playing the game, I had a strong curiosity to try different levels or tasks of a game,” “I maintained an optimistic attitude when striving to level up or complete tasks,” and “I could stay calm when striving to level up or complete a task” (Yeh et al., 2019).

The Inventory of Flow Experience in Digital Games (IFE-DG) measured the participants’ flow experience during game play. The IFE-DG includes two factors: confidence and concentration (5 items) as well as fun and challenge (4 items). EFA revealed that the factor loadings ranged from 0.682 to 0.901, and 72.58% of the total variance was explained by the two factors. The test items includes statements such as “I could concentrate on the tasks in games” and “I had a lot of fun during the game playing.” The Cronbach’s *α* coefficients were 0.914, 0.885, and 0.857 for the IFE-DG and the two factors. Moreover, the CFA indicated that the IFE-DG had good construct validity and reliability, *χ*^2^(*N* = 176, df = 64) = 149.474 (*p* < 0.05). Additionally, the GFI = 0.884, AGFI = 0.836, RMR = 0.095, and RMSEA = 0.087 (Yeh and Lin, 2018).

The Inventory of Self-Efficacy in Creativity Digital Games (IS-CDG) measured the participants’ level of self-efficacy after playing creative games. The IS-CDG includes two factors: ability to generate creative ideas (6 items) and achievement of creative performance (3 items). EFA revealed that the factor loadings ranged from 0.606 to 0.879, and 73.27% of the total variance was explained by the two factors. The test items included statements such as “I believe that I can come up with many creative ideas” and “I believe that I can be a creative person.” The Cronbach’s *α* coefficients were 0.927, 0.908, and 0.844 for the IS-CDG and the two factors. Moreover, the CFA indicated that the IS-CDG had good construct validity and reliability, *χ*^2^(*N* = 176, df = 26) = 64.113 (*p* < 0.05). Additionally, the GFI = 0.929, AGFI = 0.877, RMR = 0.065, and RMSEA = 0.092 (Yeh and Lin, 2018).

The Inventory of Mastery Experience in Creativity Digital Games (IME-CDG) measured the participants’ level of mastery experience after playing creative games. The IME-CDG includes two factors: ability to solve problems (5 items) and confidence in solving problems (3 items). EFA revealed that the factor loadings ranged from 0.606 to 0.879, and 73.28% of the total variance was explained by the two factors. The test items included statements such as “I can think of solutions quickly” and “As long as I try hard, I can come up with creative solutions.” The Cronbach’s *α* coefficients were 0.903, 0.860, and 0.819 for the IME-CDG and the two factors. Moreover, the CFA indicated that the IME-CDG had good construct validity and reliability, *χ*^2^(*N* = 176, df = 18) = 48.397 (*p* < 0.05). Additionally, the GFI = 0.932, AGFI = 0.863, RMR = 0.071, and RMSEA = 0.098 (Yeh and Lin, 2018).

### Experimental Design and Procedures

**Figure 3:**
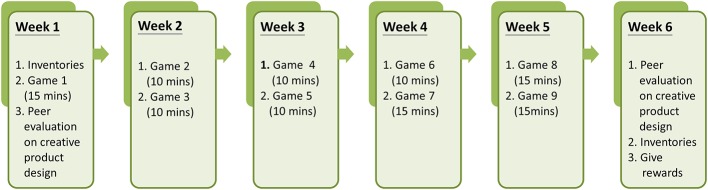
Experimental procedures.

The one-group pretest-posttest design was employed with the pretest administered in the first week, and the posttest delivered in the sixth week. The experimental instruction and all inventories conducted through the DGLC-B were designed and implemented as a part of the participants’ computer course. All experimental procedures were completed in the computer laboratory at the participants’ school. Specific experimental procedures are illustrated in Figure 3. With nine games in total, the duration of the learning program was carried out in 40 min sessions, over a total of 6 weeks.

As a method for encouraging learning motivation, participants were informed at the beginning that the games would improve their creativity and that higher value gifts would be rewarded for higher scores. The DGLC-B incorporated a variety of choices for the objects used in the creative design, task challenges varied by level of difficulty, and immediate feedback regarding the score they received was displayed at the end of each game. Notably, peer evaluations for the popularity and creativity of the designed products in game 1 and 9 were utilized to encourage idea sharing and the winners of peer evaluation were rewarded with a $5 USD gift to praise their efforts. These elements were incorporated in the procedure to increase mindful learning, flow experience, self-efficacy, and mastery experience in creativity.

To examine the instructional effectiveness, two self-evaluation questions regarding the overall impression of one’s ability and confidence of creativity were employed before and after the game playing. The self-evaluation questions were “How would you evaluate your ability of creativity?” and “How would you evaluate your confidence of creativity?” Both questions were scored from “1” point to “10” points, representing “very weak” to “very strong.”

## Results

### Effects of Aptitude × Treatment Interaction on Self-Evaluation of Creative Ability and Confidence

**Figure 4:**
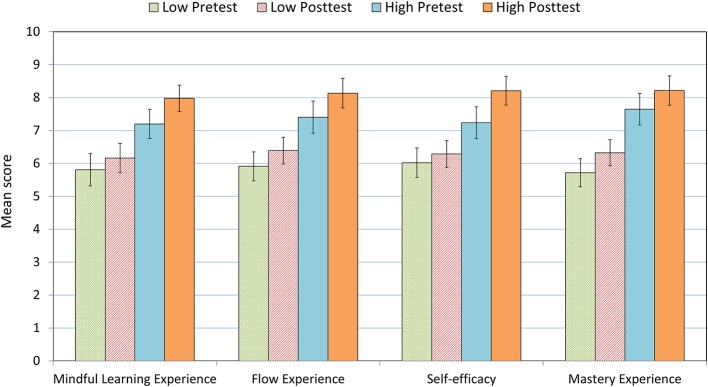
*M*s and SEs of self-evaluated creative ability in the pretest and the posttest for the low- and the high-score group of the four aptitude variables examined.

**Figure 5:**
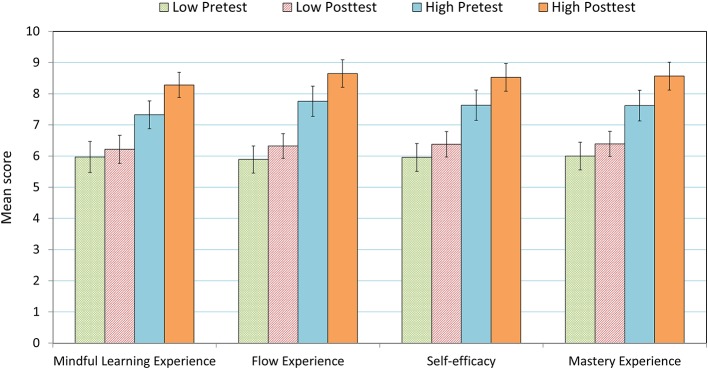
*M*s and SEs of self-evaluated creativity confidence in the pretest and the posttest for the low- and the high-score group of the four aptitude variables examined.

Using Repeated Measure Analysis of ANOVA, we examined whether there were aptitude (Group: Low vs. High) × treatment (Test: Pretest vs. Posttest) interaction effects on the participants’ self-evaluation of creative ability and creative confidence. The aptitude variables included in this study were mindful learning experience, flow experience, self-efficacy, and mastery experience; each of the independent variables was split at the median into two groups (Low vs. High). Figures 4, 5 depict the *M*s and SEs of creative ability and creative confidence for the low- and the high-score group of the aptitude variables examined.

The results showed that mindful learning experience had significant effects on changes of both creative ability and creative confidence, *F*(1, 81) = 7.152, *p* = 0.009, $\eta_{p}^{2}$ = 0.081 and *F*(1, 81) = 7.938, *p* = 0.006, $\eta_{p}^{2}$ = 0.089. Moreover, the main effects of flow experience on changes of the two creativity self-evaluations were significant, *F*(1, 81) = 7.325, *p* = 0.008, $\eta_{p}^{2}$ = 0.083 and *F*(1, 81) = 12.521, *p* = 0.001, $\eta_{p}^{2}$ = 0.134. The main effects of self-efficacy on changes of the two creativity indices were significant as well, *F*(1, 81) = 6.876, *p* = 0.010, $\eta_{p}^{2}$ = 0.078 and *F*(1, 81) = 10.235, *p* = 0.002, $\eta_{p}^{2}$ = 0.112. Also, the main effects of mastery experience on changes of creative ability and creative confidence were significant, *F*(1, 81) = 10.591, *p* = 0.002, $\eta_{p}^{2}$ = 0.116 and *F*(1, 81) = 10.022, *p* = 0.002, $\eta_{p}^{2}$ = 0.110 (see Table 1). The results revealed that pupils with a higher level of mindful learning, flow experience, self-efficacy, and mastery experience improved more creative ability and confidence than their counterparts.

**Table 1:** The effects of mindful learning, flow experience, self-efficacy, and mastery experience on self-evaluation of creative ability and confidence.

Source	ANOVA				Comparison
	*Ms*	*F*(1, 81)	*p*	$\eta_{p}^{2}$	
**Mindful learning experience**					
Ability	105.053	7.152^**^	0.009	0.081	2 > 1
Confidence	119.889	7.938^**^	0.006	0.089	2 > 1
**Flow experience**					
Ability	107.379	7.325^**^	0.008	0.083	2 > 1
Confidence	179.832	12.521^***^	0.001	0.134	2 > 1
**Self-efficacy**					
Ability	101.324	6.876^**^	0.010	0.078	2 > 1
Confidence	150.679	10.235^**^	0.002	0.112	2 > 1
**Mastery experience**					
Ability	149.725	10.591^**^	0.002	0.116	2 > 1
Confidence	147.889	10.022^**^	0.002	0.110	2 > 1

*1 = low-score group of the independent variable; 2 = high-score group of the independent variable*.

* p < 0.05;

** p < 0.01;

*** *p < 0.001*.

### The Path Model of Mindful Learning, Flow Experience, Self-Efficacy, and Mastery Experience

#### Results of the Proposed Model

Structural Equation Modeling conducted through AMOS 21 was employed to test the proposed model. In the proposed model, we hypothesized that mindful learning experience would influence mastery experience through flow experience and self-efficacy during the game-based creativity learning. The absolute fit measures suggested that the model was not a good-fit model: *χ*^2^(*N* = 83, df = 21) = 116.133, *p* < 0.001. The goodness-of-fit index (GFI = 0.815), the adjusted goodness of fit index (AGFI = 0.603), and the root mean square residual (RMR = 0.118) indicated that the proposed model was not a good-fit model. In terms of relative fit measures, the normed fit index (NFI = 0.917), the relative fit index (RFI = 0.858), the incremental fit index (IFI = 0.931) and the comparative fit index (CFI = 0.930) revealed that the model was acceptable. Finally, with regard to the parsimonious fit measures, the results of parsimony normed fit index (PNFI = 0.535) and the parsimonious comparative fit index (PCFI = 0.543) were acceptable (see Table 2). Overall, the analytical results showed that the proposed model was not a good-fit model and the direct influence of flow on mastery experience as well as that of mindful learning on self-efficacy were not significant. We therefore revised the proposed model based on results of modification indices.

**Table 2:** Direct, indirect, and total effects of the revised model.

Paths between variables	Direct effect	Indirect effect	Total effect
Mindful learning → Mastery experience	0.69	0.21	0.90
Mindful learning → Flow experience	0.91		0.91
Flow experience → Self-efficacy	0.89		0.89
Efficacy → Mastery experience	0.26		0.26
Mindful learning → Self-efficacy		0.81	0.81
Flow → Mastery experience		0.23	0.23

#### Results of the Revised Model

**Figure 6:**
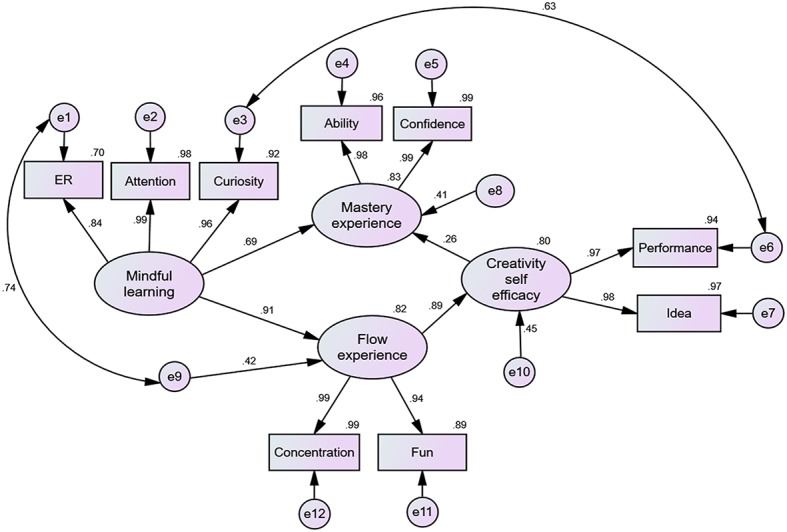
Results of the revised path model—mindfulness as an antecedent.

The important values of the revised model are shown in Figure 6. The *χ*^2^(*N* = 83, df = 21) = 42.269, *p* = 0.004; the GFI = 0.905, AGFI = 0.796, RMR = 0.040. These absolute fit measures indicated that the revised model was an acceptable model. The relative fit measures (NFI = 0.970, RFI = 0.948, IFI = 0.985, and CFI = 0.984) revealed that the model was acceptable. Finally, the parsimonious fit measures (PNFI = 0.566 and PCFI = 0.574) were also acceptable (see Table 2). In addition, all direct effects were significant (*p*s < 0.01). The standardized regression weights of all observed variables ranged from 0.84 to 0.99, *p*s < 0.01, suggesting that the revised model had a good fit of internal structure.

The standardized direct effects, indirect effects, and total direct effects of the latent variables are depicted in Table 2. With regard to direct effects, mindful learning had a stronger direct influence on flow experience than on mastery experience (see Table 2). In addition, explained variance of flow experience was 0.82, that of self-efficacy was 0.80, and that of mastery experience was 0.83 (see Figure 6); explained variance of all observed variables ranged from 0.70 to 0.99. Notably, although there were indirect influences through flow experience and self-efficacy, mindful learning and flow experience were the most powerful direct predictors of mastery experience during game-based creativity learning.

## Discussion

This study developed a 6-week game-based experimental creativity learning program (DGLC-B) for pupils in the 5th and 6th grades, and further explored the relationships among mindful learning experience, flow experience, self-efficacy, and mastery experience. The results suggest that the DGLC-B is effective in enhancing 5th and 6th graders’ self-perceived creative ability and confidence. The DGLC-B is a comprehensive and original creativity enhancement program within the field of game-based learning; it incorporates the training of dispositions and strategies of creativity, as well as a variety of divergent and convergent thinking skills which are pertinent to creativity learning. Notably, the DGLC-B includes creative product design which require creativity heuristics. In addition, peer evaluation of other participants’ product design was utilized as a type of idea sharing and inspiration. This method of boosting creativity falls in line with past findings concluding that participants who were exposed to the ideas of others had improved creativity (Fink et al., 2010).

In a meta-analysis of 70 studies concerning creativity training, Scott et al. (2004) claimed that the most successful programs “were likely to focus on development of cognitive skills and the heuristics involved in skill application, using realistic exercises appropriate to the domain at hand” (p. 361). In agreement with this conclusion, the DGLC-B incorporated the following items to enhance mindful learning, flow experience, self-efficacy, and mastery experience: appropriately challenging tasks, object choices for creative design, immediate feedback regarding performance of each game, idea sharing through peer evaluations for designed products, and gifts for high performance quality. Results suggest that pupils with a higher level of mindful learning, flow experience, self-efficacy, and mastery experience improved more in their creative ability and confidence than their lower scoring peers. The findings of the present study reinforce the effectiveness of our design for the DGLC-B, in addition to other research advising that appropriately challenging tasks are pertinent for increasing flow experience in children (Inal and Cagiltay, 2007), as well as that the positive effect of applying games for desired learning outcomes and pleasurable engagement (Hamari et al., 2016; Hawlitschek and Joeckel, 2017).

The major hypothesis proposed by the current researchers was that mindful learning would directly influence mastery experience and self-efficacy, as well as indirectly influence mastery experience through flow experience and self-efficacy within this game-based creativity training. In addition, the researchers hypothesized that flow experience would carry effects on mastery experience; however the path model analysis did not support the direct influence of flow experience on mastery experience or mindful learning and self-efficacy. Rather, mindful learning influenced mastery experience through flow experience and self-efficacy. Interestingly, this study found that mindful learning experience had a stronger direct influence on flow experience than on mastery experience. Although there were indirect influences through flow experience and self-efficacy, mindful learning remained the most powerful direct predictor of mastery experience during game-based creativity learning. These findings highlight the critical role of mindful learning and flow experience during game-based creativity training.

The important argument here is that game-based learning may foster mindful learning by catching the full attention of players which results in enhanced learning experience (Langer, 2016). In game playing, mastery experiences, or the “win” experiences, are a vital part of video games (Starks, 2014). Digital games can attract learners’ attention and encourage repeated exposure to recommended behaviors or skills (Cohen, 2016). Therefore, a well-designed creativity game is likely to increase mastery experience of creativity. When learning mindfully, the learner (1) actively notices and is open to new things within the present context, (2) utilizes cognitive flexibility, (3) shows perseverance in problem solving, (4) regulates emotions, and (5) channels this thinking process by taking multiple perspectives to find many solutions that fit the context (Langer, 1993; Davenport and Pagnini, 2016; Bercovitz et al., 2017). These characteristics of mindful learning undoubtedly contribute to the learning of creativity. It has been suggested that the mind can control flow, and that mindfulness training can enhance self-regulation, and facilitate flow states for optimal performance (Kaufman et al., 2018). These findings support our results that mindful learning has strong influences on flow experience which can lead to enhanced learning and exploratory behavior (Skadberg and Kimmel, 2004; Kiili, 2005; Hamari et al., 2016; Hawlitschek and Joeckel, 2017), and improve self-efficacy, motivation, and fun or the feeling of happiness (Starks, 2014; Ribera and Ceja, 2018). Moreover, this study found that self-efficacy works as a mediator between flow experience and mastery experience, which highlights the contribution of self-efficacy for perseverance and resiliency when attaining desired outcomes (Bandura, 2012). Self-efficacy is additionally a mediator of creativity performance (Liao et al., 2010; Wang et al., 2018).

To integrate this collection of data, mindful learning experience leads to a stronger sense of self-efficacy and mastery experience through flow experience during game playing. These phenomena can present themselves in a positive cycle, where increased flow experience enhances positive self-efficacy and perceived mastery experience. To conclude, with the clear association between mindful learning, flow experience, self-efficacy, and mastery experience, mindful learning is necessary in the achievement of mastery experience in game-based creativity training.

## Conclusion

Past studies suggest that digital game-based learning can be a promising tool for improving creativity. However, few game-based learning programs for creativity have covered comprehensive creativity training strategies, including diverse creativity dispositions and skills. This study aimed at developing such a game-based creativity learning program for pupils in late elementary school. The Digital Game-based Learning of Creativity (DGLC-B) allowed for exploration into how mindful learning could influence mastery experience within digital creativity games, and the potential mediation of flow and creative self-efficacy. The empirical evidence found in this study suggests that the concerned personal traits interact with the treatment employed in the game-based program; pupils with higher levels of these personal traits improve more in self-perceived creative ability and confidence upon the completion of the training program. In addition, mindful learning experience is critical to the achievement of mastery experience during game-based creativity learning; it carries both direct and indirect influence on mastery experience through flow experience and self-efficacy of creativity.

This study contributes to the theoretical construction of how game-based learning can be designed to facilitate mindful learning experience, flow experience, self-efficacy, and mastery experience during creativity training among pupils. Effective mechanisms include providing rewards for high-quality performance, challenging tasks, free choices of design components, immediate feedback, and idea sharing. The profound theoretical framework proposed in this study provides a valuable approach for creativity instruction through game-based learning or classroom instruction.

## Limitations and Suggestions

Owing to the lengthy experimental instructional period (6 weeks) and the limited number of desktops within the computer class (only one class per week) in elementary schools, it was challenging to recruit a significant sample for this experimental instruction. Further studies can include a bigger sample to replicate the proposed model in this study. However, the participants were positive about the game-based creativity program. The results also show positive learning outcomes. Further studies may adapt the designs of this program in classroom instruction to enhance pupils’ creativity.

Langarian mindful learning emphasizes the continuous creation of new categories, openness to new information, and an implicit awareness of more than one perspective (Langer, 2016, p. 4). Based on these concepts, Yeh et al. (2019) developed a mindful learning inventory for game-based learning which additionally includes grit and emotional regulation. This study employed Yeh et al’s inventory and found such mindful learning is powerful in predicting learning outcomes concerning creativity, suggesting the importance of including the components of grit and emotional regulation in mindful learning. Furthermore, mindful learning interventions within classrooms have been found to be effective in improving pupils’ learning of diverse subjects (Bakosh et al., 2016) as well as in self-evaluation (Malboeuf-Hurtubise et al., 2018). This study also found that enhancing mindful learning experience is valuable for helping pupils to achieve flow experience, and to be more confident and competent in the application of creativity dispositions and strategies. This study incorporated positive feedback, rewards, peer evaluation, idea sharing, and product design through computers only. Further studies may integrate face-to-face discussion after each training session to maximize the learning effect.

Finally, flow experience can be enhanced by matching difficulty level to ability, providing clear goals, unambiguous feedback, concentration on the task at hand, action-awareness merging, sense of control, and intrinsic reward (Swann et al., 2012; Kaufman et al., 2018). Our game-based learning program incorporated most of these mechanisms and found that flow experience was a powerful predictor of self-efficacy and mastery experience during the game-based training of creativity. Accordingly, these mechanisms should be considered when designing a game-based learning program for pupils.

## Data Availability

The datasets for this study can be found in “mindful data available” (https://drive.google.com/file/d/1VPJa3WwgbGiNNsRZ2vmupylLwd9jeEU_/view?usp=sharing).

## Ethics Statement

This study was carried out in accordance with the recommendations of Research Ethics Committee of National Chengchi Universtity with written informed consent from all subjects. All subjects gave written informed consent in accordance with the Declaration of Helsinki. The protocol was approved by the Research Ethics Committee of National Chengchi Universtity.

## Author Contributions

YY conceived the theoretical conceptualization and designed the study, analyzed the data, and wrote the manuscript. S-YC analyzed the data, and contributed to the writing of the manuscript. ER contributed to the writing of the manuscript. C-SL contributed to the collection of data.

### Conflict of Interest Statement

The authors declare that the research was conducted in the absence of any commercial or financial relationships that could be construed as a potential conflict of interest.
